# Development of the ultrastructure of sonic muscles: a kind of neoteny?

**DOI:** 10.1186/1471-2148-14-24

**Published:** 2014-02-07

**Authors:** Sandie Millot, Eric Parmentier

**Affiliations:** 1 Laboratoire de Morphologie Fonctionnelle et Evolutive, Institut de Chimie, B6C, 4000 Liège, Belgium

**Keywords:** Piranha, Call, Development, *Pygocentrus*, Sonic muscle

## Abstract

**Background:**

Drumming muscles of some sound-producing fish are ‘champions’ of contraction speed, their rate setting the fundamental frequency. In the piranha, contraction of these muscles at 150 Hz drives a sound at the same frequency. Drumming muscles of different not closely related species show evolutionary convergences. Interestingly, some characters of sonic muscles can also be found in the trunk muscles of newly hatched larvae that are able to maintain tail beat frequencies up to 100 Hz. The aim of this work was to study the development of sound production and sonic and epaxial muscles simultaneously in the red bellied piranhas (*Pygocentrus nattereri*) to seek for possible common characteristics.

**Results:**

Call, pulse and period durations increased significantly with the fish size, but the call dominant frequencies decreased, and the number of pulses and the call amplitude formed a bell curve. In epaxial muscles, the fibre diameters of younger fish are first positioned in the graphical slope corresponding to sonic muscles, before diverging. The fibre diameter of older fish trunk muscles was bigger, and the area of the myofibrils was larger than in sonic muscles. Moreover, in two of the biggest fish, the sonic muscles were invaded by fat cells and the sonic muscle ultrastructure was similar to the epaxial one. These two fish were also unable to produce any sound, meaning they lost their ability to contract quickly.

**Conclusions:**

The volume occupied by myofibrils determines the force of contraction, the volume of sarcoplasmic reticulum sets the contraction frequency, and the volume of mitochondria sets the level of sustained performance. The functional outcomes in muscles are all attributable to shifts in the proportions of those structures. A single delay in the development restricts the quantity of myofibrils, maintains a high proportion of space in the sarcoplasm and develops sarcoplasmic reticulum. High-speed sonic muscles could thus be skeletal muscles with delayed development. This hypothesis has the advantage that it could easily explain why high-speed sonic muscles have evolved so many times in different lineages.

## Background

Fish species have developed different mechanisms allowing them to produce sounds. Those involving the use of fast sound producing muscles [1,2] and swim bladders can be roughly divided into four groups: 1) those whose muscles are directly inserted on areas covering important parts of the swimbladder, such as in the toadfish taxa [3-6], the searobin [7] or pimelodids [8,9]; 2) those whose muscles possess ventral tendons running from the left to the right sonic muscles, such as in piranhas [10-12], 3) those whose muscles lie on the body wall and extend nearly the entire length of the swimbladder on which muscles fibers are inserted at the level of a dorsal aponeurotic sheet, such as in the weakfish [13,14] or the meagre [15] and 4) those muscles insert on a thin bony plate that attaches to the swimbladder (elastic spring mechanism), such as in doradids, mochokids and ariids [9,16,17].

In these drumming fishes, the muscle contraction rate sets the fundamental frequency [12,18-21]; *i.e.* contraction of sonic muscles at 200 Hz will drive a harmonic sound with a fundamental frequency of 200 Hz. It implies that the time frame to perform a contraction/relaxation cycle is very short: toadfish sonic muscles require about 10 ms for a twitch [4,18] and the EMGs of weakfish sonic muscle twitches range from 7.9–13.6 ms in duration [22].

The origin of drumming muscles differs among families. In Batrachoidiforms, Siluriforms or Scorpaeniforms the common innervations of the sonic muscle by occipital nerve roots suggests the drumming muscles derive from occipital somites [23-26]. However, according to Ladich and Bass [11], in piranhas (characiform) it seems unlikely that sonic muscles derive from occipital somites because their innervation is accomplished by true spinal nerves.

Fibers have a number of morphological and biochemical convergent adaptations for speed [27-30]. Muscles with a brief activation-relaxation cycle require a potential increase in the volume of the Sarcoplasmic Reticulum (SR) and mitochondria, which reduces the space available for the force-generating myofilaments [28,31,32].

In addition, many of these muscles have an unusual radial morphology in which the contractile cylinder comprises alternating ribbons of SR and myofibrils. In some species, such as the toadfish *Opsanus tau* or the catfish *Platydoras armatulus,* a central core of sarcoplasm can be found [9,29]. This arrangement would be an adaptation for speed because it minimizes travel distance for calcium between the SR and myofibrils. According to the synthesis of Ladich and Fine [33], these muscles would also have the fastest calcium spike in a vertebrate muscle [34], rapid cross-bridge detachment [35], huge activator stores of calcium [36,37], multiple innervations of muscle fibers [38,39] and a different component distribution of parvalbumins [40].

According to the literature, rapid contractions have also been observed in other fish muscles. During cyclic swimming, three-day old zebrafish larvae were able to maintain tail beat frequencies up to 100 Hz [41,42]. The same kind of observation (Mauguit, pers.com.) has been made during the development of swimming abilities in the catfish *Corydoras aeneus*[43]. Such unusual muscle contraction speed is close to the one of different sound-producing muscles, such as in the piranha for instance [10,44]. We expected to discover that larval swimming muscles share some of the special adaptations found in fast synchronous muscles similar to the findings of Müller and van Leeuwen [41]. In just hatched larvae of *Danio rerio* and *Corydoras aeneus*, a central core of sarcoplasm surrounded by a tubular contractile apparatus can be found in white fibers [45] like in some sonic muscles [25,30,46]. Fish larvae only generate these very high tail beat frequencies during the first few days after hatching: maximum tail beat frequency drops rapidly with age. Consequently, the decreasing tail beat frequencies in the first weeks of larval development should correspond to the rapid increase of myofibril contents in trunk muscles.

The aim of this work was simultaneously to study the development of sound production and sonic and epaxial muscles in red bellied piranha. We hypothesized that high speed muscles could be the result of delayed skeletal muscle development, restricting the quantity of myofibrils, maintaining a high proportion of space in the sarcoplasm and developing sarcoplasmic reticulum.

## Results

### Acoustical characteristics of sound produced by fish from each size class

**Figure 1:**
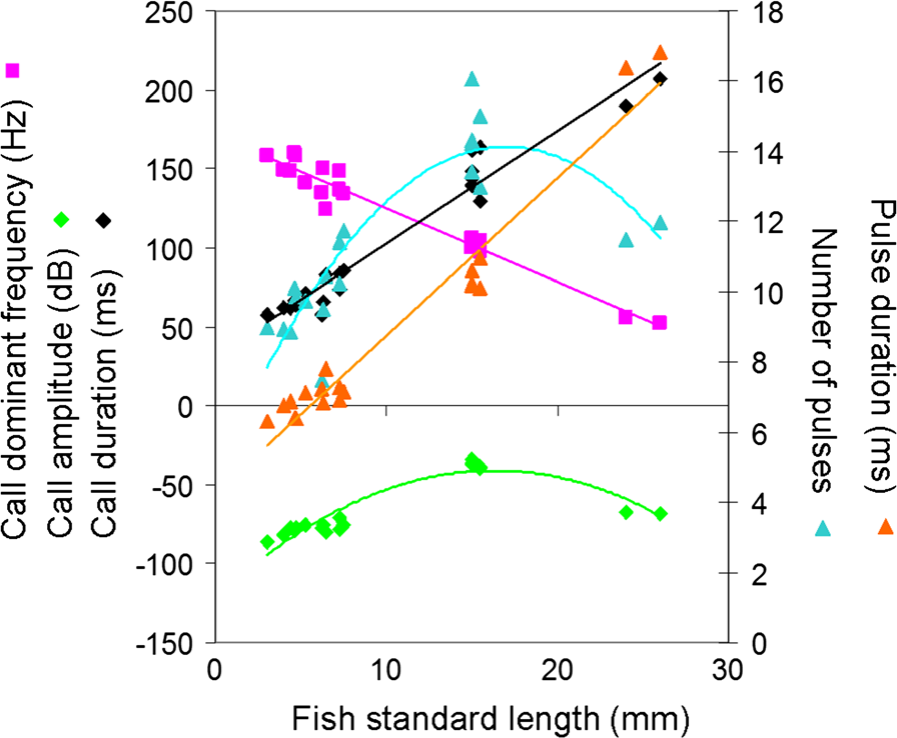
**Acoustical characteristics of sounds produced by *Pygoncetrus nattereri *of different sizes.** The straight lines correspond to linear regression and the curve lines to polynomial regression.

Sounds were successfully recorded for all fish from size classes 3 to 5, but unexpectedly two fish from size class 5 were not able to produce sounds. Call and pulse durations increased significantly with fish size (F_2,17_ = 177.80, p < 0.001; F_2,17_ = 569.09, p < 0.001 respectively). The call dominant frequency decreased significantly with fish size (F_2,17_ = 106.85, p < 0.001). The number of pulses and the call amplitude were significantly higher for size class 3 than for size classes 4 and 5 (F_2,17_ = 33.93, p < 0.001; F_2,17_ = 322.69, p < 0.001 respectively; Table 1; Figure 1).

**Table 1:** Mean ± SD of call and pulse duration (ms), call dominant frequency (Hz), call amplitude (dB) and number of pulses per call for each fish size class

**Fish size class**	**Call duration (ms)**	**Pulse duration (ms)**	**Dominant frequency (Hz)**	**Number of pulses**	**Call amplitude (dB)**
3	69.4 ± 9.9	7 ± 0.5	145.26 ± 11.2	9.8 ± 1.2	-77.7 ± 3.7
4	147.8 ± 13.0	10 ± 0.3	102.2 ± 3.1	14.3 ± 1.1	-37.1 ± 2.1
5	198.5 ± 12.2	16.6 ± 0.3	54.1 ± 2.3	11.7 ± 0.3	-67.6 ± 0.2

### Structure and ultrastructure of epaxial and sonic muscles for fish from each size class

**Figure 2:**
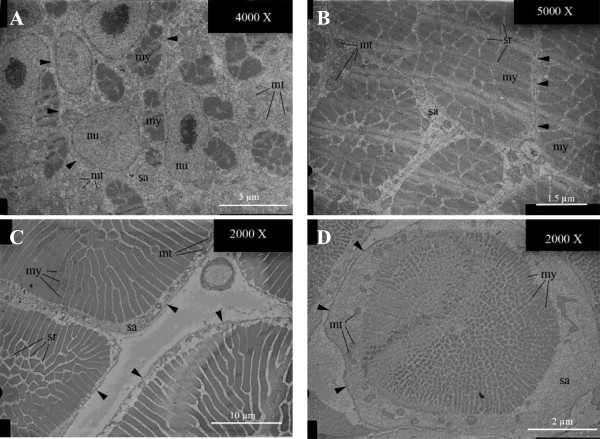
**Ultrastructure of epaxial and sonic muscles in *Pygocentrus nattereri *of different sizes.** Ultrastructure of epaxial muscle in *Pygocentrus nattereri* of 3 mm **(A)**, 25 mm **(B)** and 146 mm **(C)** length and ultrasturcture of sonic muscle from fish of 146 mm **(D)** length. mt: mitochondria, my: myofibril, nu: nucleus, sa: sarcoplasm, sr: sarcoplasmic reticulum, arrow head: sarcolem.

In size class 1 (fish of 3 mm length), epaxial cell muscles possessed a small diameter (7.84 ± 0.8 μm, n = 7). At this stage, the cell space was mainly occupied by the nucleus, and the ultrastructure was characterized by scarce myofibrillar packs as small as mitochondria (Figure 2A). The sarcoplasmic reticulum was not well developed. In older fish (size class 2; length of 25 mm), the number of myofibril packs increased and was concentrated in the centre of the cell (Figure 2B). A layer of sarcoplasm had developed around the myofibril packs and contained many mitochondria. In the next stages (size classes 3 and 4; from length of 50 mm to 150 mm), the external packs of myofibrils elongated, and the outer ring of sarcoplasm shortened (Figure 2C), being narrowest in larger fish. In the centre of the cell, myofibrils were more rounded.

**Figure 3:**
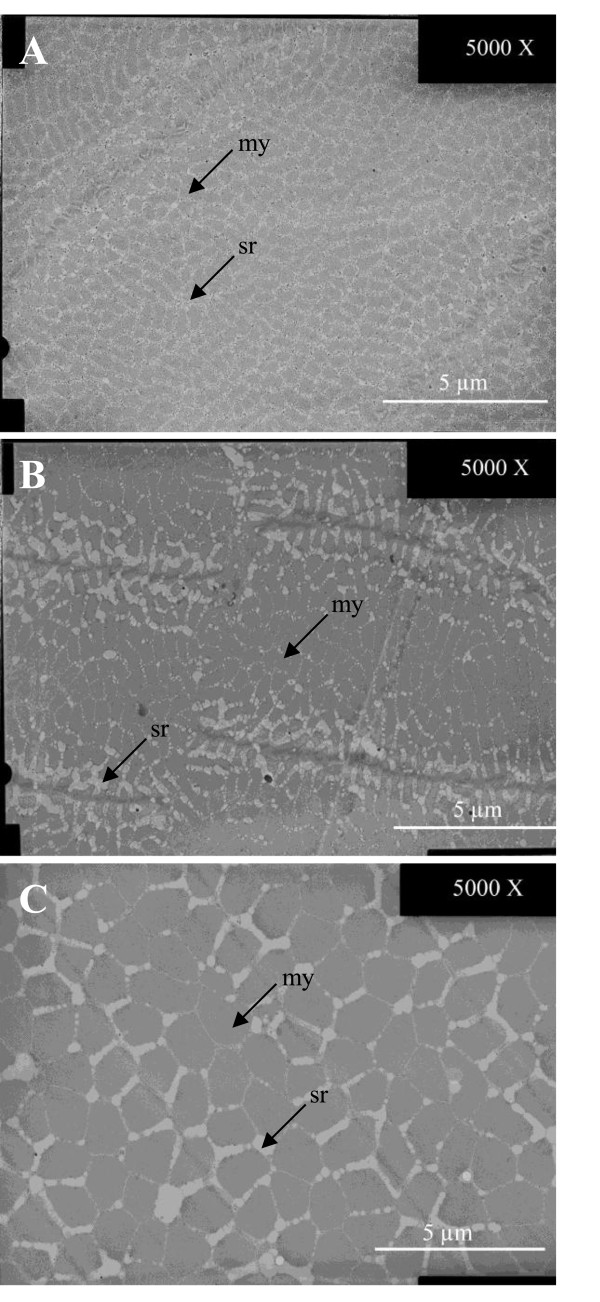
**Ultrastructure of sonic and expaxial muscles in *Pygocentrus nattereri *of size class 5 (245 mm length). (A)** sonic muscle of a calling fish; **(B)** sonic muscle of a mute fish and **(C)** epaxial muscle of a calling fish. my: myofibril, sr: sarcoplasmic reticulum.

Sonic muscles were not found in fish of 3 mm and 25 mm length. In the different older fish, the general ultrastructure of the sonic muscle (Figure 2D) was similar and corresponded to the description given by Eichelberg [47]. Each fiber contained a core of myofibrils forming small patches separated by an irregular arrangement of sarcoplamic reticulum, which was particularly abundant and formed vesicles. Moreover, in some cases the core of myofibrils had in its centre hollow spaces of sarcoplasm. On the periphery, there was a space between the sarcolemma and the core of myofibrils. Numerous mitochondria were located in this peripheral space, which was always larger than in the epaxial muscles of fish from the same size (Figure 2D). The differences between the sonic muscles were mainly found in the size of the cell, the thickness of the outer band of sarcoplasm on the periphery and the proportion of myofibrils and sarcoplasmic reticulum. In the biggest fishes (size class 5; length of 245 mm) the sonic muscles of two kinds of individuals (calling and muted fish) were compared. Calling fish still had the same kind of morphology that was previously described (Figure 3A). Mute fish show different modifications. First, the sonic muscle was invaded by fat cells. Second, at the level of the ultrastructure the outer ring of sarcoplasm had disappeared (Figure 3B). Moreover, the organization of the myofibrils and sarcoplasmic reticulum was a combination of those observed in the sonic and epaxial muscles of calling fish (Figure 3A, C). Indeed, myofibril packs of mute fish were still smaller than in epaxial muscles, but the space between these packs were larger than in the sonic muscle of calling fish. Moreover, the sarcoplasmic reticulum seemed to be less structured in muted fish (Figure 3B).

### Fiber diameter of sonic and epaxial muscles for fish from each size class

**Figure 4:**
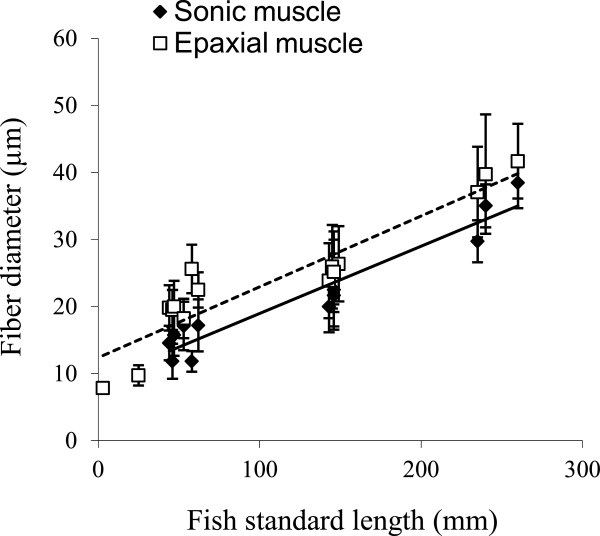
**Fiber diameter (Mean ± SD) of sonic and epaxial muscles in *Pygocentrus nattereri *of different standard length.** The black line represents the linear regression for sonic muscle and the dotted line represents the linear regression for the epaxial muscle. Note that epaxial muscle diameter of younger fish (3 and 25 mm) are on the same slope as sonic muscles.

In fish size classes, when the comparison was possible (all except size classes 1 and 2), the fiber diameter of epaxial muscle was always higher than the fiber diameter of sonic muscle (F_2,22_ = 27.62, p < 0.001; Figure 4). In fish from size class 3 to 5, the fiber diameter of sonic and epaxial muscle significantly increased with the fish standard length (F_2,11_ = 58.09, p < 0.001; F_3,12_ = 146.73, p < 0.001 respectively; Figure 4). The positioning of data from the trunk muscle of younger fish (size classes 1 and 2) was interesting because they were positioned on the slope of the sonic muscles and not of the epaxial muscles. There seemed to be an acceleration in the growth of the epaxial muscles, but the sonic muscles, which are derived from hypaxial muscles, were still developing at the same rate.

### Proportion of space devoted to myofibrils in sonic and epaxial muscles

**Figure 5:**
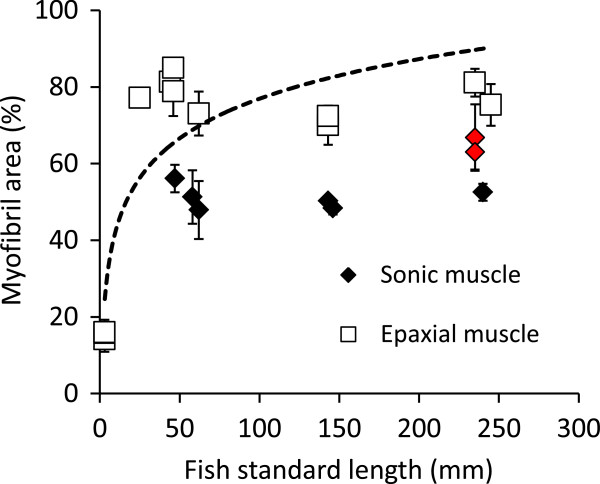
**Proportion of myofibril areas (Mean ± SD; in %) in sonic and epaxial muscle fiber in *Pygocentrus nattereri *of different sizes.** The dotted line corresponds to the logarithm tendency for the epaxial muscle. The red dots represent the proportion of myofibril in the sonic muscle of mute fish in size class 5.

In size classes 3 to 5, the proportion of myofibrils in the cross section of epaxial muscle was always higher than for the sonic muscle (F_1,56_ = 135.22, p < 0.001; Figure 5). It was significantly lower in fish from size class 1 than in fish from the other size classes. This proportion was also lower for fish from size class 4 compared to fish from size classes 3 and 5 (F_4,34_ = 235. 95, p < 0.001; Figure 5). The proportion of myofibrils in cells of sonic muscle was significantly higher for mute fish than for calling fish of size class 5 or for fish from the other size classes (F_3,25_ = 11.65, p < 0.001; Figure 5).

## Discussion

Call duration, which averaged approximately 70 ms in 50 mm long specimens, increased to 200 ms for fish over 250 mm. This increase appeared mainly to be due to longer pulses. In contrast, fish growth was related to a simultaneous decrease in the fundamental frequency. The impact of fish size on spectral and some temporal features such as pulse duration is well-known in distantly related fish families [21,48-53]. The slope of this relationship is, however, generally less sizeable in fish whose sound production is based on a forced response of the swim bladder by the high-speed contraction of sonic muscles. In piranha, the calling frequency of 50 mm specimens was 150 Hz, compared to 60 Hz in a five times longer specimen (245 mm). For comparison, the calling frequency of a 60 mm clownfish (whose mechanism is not based on a forced response) is 700 Hz, but that of a 130 mm specimen, which is only two times longer, is less than 400 Hz [51]. In other words, the high slope value of the correlation between fish size and dominant frequency in the clownfish indicates that the size of the emitter can be assessed by the receiver and so be used in sonic communication. In the grunt of the gurnard *Eutrigla gurnardus*[21], in the weakfish *Cynoscion regalis*[23,50], in the toadfish *Halobatrachus didactylus*[54], in the holocentrids [55], and in *Pygocentrus nattereri*, this kind of relationship has also been statistically established. However, the slope value of the relationship is very weak and it is difficult to determine whether the fish can discriminate the spectral characteristic of the call as in the previous group. In fishes having the forced response of the swim bladder, the relationship between frequency and size has been explained only once [50]. This explanation is based on the scaling effect [56,57]: bigger fish have longer muscles so it would take more time to complete a muscle twitch, resulting in a longer period in the acoustic waveform and therefore a lower dominant frequency.

However, according to the variations in sound amplitude and number of pulses per call, the scaling effect may be insufficient to completely explain the relationship. On the basis of our histological data, other factors than the muscle length are linked to the muscle contraction speed and consequently to the muscle contraction rate.

In the weakfish, *Cynoscion regalis*, the sound pressure level increases with fish size. Acoustic pressure is proportional to the product of surface area and movement velocity [58]. Therefore, increasing swimbladder size in larger fish should increase sound amplitude [50]. In toadfish sounds, the amplitude increases with the vibration rate of the swimbladder walls [4]. Unlike in those species, the call amplitude is lower in larger specimens of *P. nattereri*. Because the swimbladder size usually increases proportionally to the fish size, it means the decrease in sound pressure level has to be found at the level of the muscle physiology. In the weakfish, a decrease of sound pressure level is due to muscle atrophy [13,50] and in the toadfish slow movements of the swimbladder fail to produce audible sound [4]. The low amplitude of larger piranha sounds is probably due to a reduced muscle contraction rate, which could be due to the scaling effect. However, the decrease of the muscle performance is also reflected in the lower number of pulses per call in larger specimens, and this characteristic is not due to the scaling effect.

The problem is to determine why the velocity and the ability to sustain contraction are affected. Muscles with a brief activation-relaxation cycle require a potential increase in the space devoted to the sarcoplasmic reticulum, sarcoplasm and mitochondria, all of which reduce the space available for the force-generating myofilaments [28,31,32]. The comparison between epaxial and sound-producing muscles indicated clearly that the lower ratio of myofibrils in sonic muscles is related to the capacity for high-speed contractions. Moreover, in fish from 25 mm to 245 mm in length, the measure of the ratio of myofibrils did not take into account the outer ring of sarcoplasm, meaning the myofibril area is overestimated. In the two big specimens (>230 mm) unable to make sound, the ratio of myofibrils was significantly higher than in all other sonic muscles and close to the one measured in the epaxial muscle. Moreover, the outer ring was reduced completely as it is in epaxial muscles. Fish of the same size having a lower amount of myofibril areas were still able to make sounds. In other words, it seems that the ability of fish to produce sounds is determined by the proportion of the cell components. However, the presence of fat cells in the sonic muscles of mute fish, cannot be rejected as playing an important role in the loss of the ability to produce sound.

Sciaenid sonic muscles are probably modified from hypaxial (trunk) muscles which are formed during maturity on top of the swimbladder [59], and they are serially innervated by true spinal nerves from a number of body segments [14]. According to muscle innervation, the sonic and body muscles of piranha have a common origin [60], supporting the idea that both muscles also derive from body myomeres. This common origin can explain why we did not find sound-producing muscles at the level of the anterior ribs in younger larvae (3 mm to 25 mm length). They probably had yet to differentiate from hypaxial muscle. Moreover, the development of the fiber diameter provides further information: the cell diameter of epaxial fibres in younger fish is on the same slope as the data coming from the sonic muscles (Figure 4). It seems there is acceleration in the growth of epaxial muscles, but the sonic muscles look like hypaxial muscles, developing at the same rate.

Comparing epaxial and sound-producing muscles allows us to consider that the morphology of high-speed sonic muscles could correspond to a kind of neoteny in which myofibril development is stopped before achieving the body cell morphotype. This hypothesis has the advantage that it could easily explain why high-speed sonic muscles have evolved so many times in different lineages. To support our hypothesis, literature reports that very young fish larvae are also able to make high-speed contractions. Larval muscles cells are obviously smaller than in adults and have little space devoted to myofibrils because they are in development. Some species can also show a central core of sarcoplasm surrounded by a tubular contractile apparatus [45,61,62] as is the case, for example, in the sound producing muscles of *Opsanus tau*[29,30] or of the doradids [9]. Trunk muscles, however, lose these characteristics very quickly at the beginning of development [41,42], explaining why they are already absent in 25 mm length piranhas. However, in very young larvae of piranha (1 day post hatching or 3 mm length), myofibrils occupy only 10% of the muscle cell. That is less than in the sonic muscle, meaning that sonic and epaxial muscles should share a common stage of development.

## Conclusions

The volume occupied by myofibrils determines the force of contraction, the volume of sarcoplasmic reticulum sets the contraction frequency, and the volume of mitochondria sets the level of sustained performance. The entirety of functional outcomes in muscle are all primarily attributable to shifts in the proportions (and relationships) of those three structures [2,63]. On the basis of our results we cannot argue that the size of the muscle cells and the proportion of their components are the only characteristics allowing high-speed contractions in sonic muscles. For example, many studies showed parvalbumin isoforms and myofibrillar proteins differ between fish larvae and adults [64-67], giving them different contractile properties. However, we think that the ultrastrucular features are sufficient to allow high-speed contractions. To conclude, we propose the idea that high-speed sonic muscle could be skeletal muscle that were delayed in their normal development. This assumption is supported by at least two observations: 1) the ultrastructure of the fibres of young fish and 2) the cell morphology of sonic muscles in muted fish. This hypothesis has the advantage that it could easily explain why high-speed sonic muscles have evolved so many times in different lineages.

## Methods

### Ethics

Experimental and animal care protocols followed all relevant international guidelines and were approved by the ethics commission (no. 728) of the University of Liège.

### Biological material

Piranhas of 3 mm (1 day post-hatching) and 20–25 mm (25 days post-hatching) were kindly donated to the study by private individuals. Larger specimens (40–80 mm and 140–150 mm) were purchased from a specialised store (Aqua Garden Centre, Liège, Belgium). The largest fish, from 230–260 mm, were donated by the aquarium of Liège. All fish were kept by size class in 300 L tanks. The water temperature was maintained at 26 ± 2°C and the oxygenation level above 90%. Fish were fed mussels and smelts three times a week.

### Recording and analysis of sound production

The experiment was carried out on five groups of fish that belonged to different size classes. The first group (size class 1) was composed of 7 individuals with a mean standard length of 3 mm. The second group (size class 2) was composed of 3 fish between 20 and 25 mm in length. Fish from both of these groups were too small for any sound recording (previously checked) and were directly euthanized for further morphological analyses. The three other groups were composed respectively of 12 fish with a standard length between 40 and 80 mm (size class 3), 6 fish of 140 to 150 mm (size class 4) and 4 fish of 230 to 260 mm (size class 5). In order to record sounds, each fish was transferred to a 160 L experimental tank (90 cm length × 35 cm breadth × 50 cm depth) and hand held (with a small pressure on the belly) in 5 cm distance to the hydrophone (HTI-96-MIN SERIES; High Tech inc, Mississippi, USA) in order to compare the acoustic signal between fish size classes. The characteristics of the water in the experimental tank (physico-chemical composition, temperature and oxygen level) were similar to those in the rearing tanks.

Only sounds with a good signal to noise ratio were analysed. Avisoft-SASLab pro software (Avisoft Bioacoustics, Berlin, Germany) was used for each analysis. Temporal features were measured from oscillograms, and frequency parameters were obtained from power spectra transformed with a Fast Fournier Transformation (Hamming window). The following temporal and spectral characteristics of the sound waves were measured: 1) Pulse and call duration (ms): time between the onset of one pulse or call and its end 2) Number of pulses within a sound 3) Call amplitude (dB) 4) Dominant frequency (Hz): the highest energy in the whole sound.

### Morphological study

All specimens used for the morphological study were euthanized with MS-222 (500 mg L^-1^) but were subsequently treated differently according their size. Fish from size class 1 were entirely fixed for 48 h in 2.5% glutaraldehyde for observation by transmission electron microscopy (TEM). Fish from other classes were dissected under a binocular microscope, and tissue samples (sonic muscles and white epaxial muscles) were fixed for 2 days in the 2.5% glutaraldehyde solution.

All fish and muscle samples were post-fixed in 1% osmium tetroxide, dehydrated through a graded ethanol-propylene oxide series and embedded in epoxy resin (SPI-PON 812, SPI-CHEM, Leuven, Belgium). Semithin sections (1 μm) and ultrathin sections (60–80 nm) were cut using a diamond knife on a Reichert Ultracut E ultramicrotome. Toluidine blue-stained semithin sections were used for general histology and for orientation to target the area of further ultrathin sections. They were observed and photographed with a Leica MD 1000 binocular microscope equipped with a digital camera (Canon Power Shot S50, Diegem, Belgium). Ultrathin sections were classically stained with uranyl acetate and lead citrate, then viewed in a JEOL JEM 100SX transmission electron microscope (Zaventem, Belgium) at 80 kV accelerating voltage.

The fiber diameters were measured on semithin sections. Pictures from electron microscopy were used to reveal the muscle morphological structures and to determine the ratio of the surface of sarcoplasmic reticulum and myofibrils in the muscle cells. Using Adobe Photoshop, the total number of pixels was first determined on digitalized pictures. A layer was then applied to the picture, and the myofibrils or sarcoplasmic reticulum were stained. The number of pixels corresponding to the stained surfaces was then used to calculate the ratio.

### Data analysis

Statistical analyses were performed using Statistica 7 software (Statsoft, USA). The results were expressed as mean ± standard deviation (SD). Data were checked for normality with the Shapiro-Wilk test. The data all complied with parametric tests to be used. One-way ANOVA were used to analyse differences in call and pulse duration, number of pulses, call amplitude and dominant frequency for the three fish size classes for which the sound recording was possible (size classes 3, 4 and 5). One-way ANOVA were used to analyse differences in fiber diameter and fiber proportion of myofibrils between fish from each size class for sonic and for epaxial muscles. Homogeneous groups were determined with an *a posteriori* Newman and Keuls test. For all tests, the significant threshold was p < 0.05.

## Competing interests

The authors declare that they have no competing interests.

## Authors’ contributions

SM and EP conceived and designed the experiments, SM performed the recording experiments, EP realized the microscopy. SM and EP analyzed the data and wrote the paper. All authors read and approved the final manuscript.

## References

[B1] Tavolga WN Tavolga WN Sonic characteristics and mechanisms in marine fishes Marine Bio-acoustics 1964 Oxford: Pergamon Press 195 211

[B2] Rome LC Linstedt SL The quest for speed: muscles built for high-frequency contractions News Physiol Sci 1998 13 26 268 11390801 10.1152/physiologyonline.1998.13.6.261

[B3] Tower RW The production of sound in the drumfishes, the searobin and the toadfish Ann N Y Acad Sci 1908 18 149 180 10.1111/j.1749-6632.1908.tb55101.x

[B4] Fine ML Malloy KL King CB Mitchell SL Cameron TM Movement and soung generation by toadfish swimbladder J Comp Physiol A 2001 187 371 379 10.1007/s003590100209 11529481

[B5] Rice AN Bass AH Novel vocal repertoire and paired swimbladders of the three-spined toadfish, *Batrachomoeus trispinosus*: insights into the diversity of the Batrachoididae J Exp Biol 2009 212 9 1377 1391 10.1242/jeb.028506 19376959 PMC2726849

[B6] Fine ML King CB Cameron TM Acoustical properties of the swimbladder in the oyster toadfish *Opsanus tau* J Exp Biol 2009 212 21 3542 3552 10.1242/jeb.033423 19837896 PMC2762879

[B7] Connaughton MA Sound generation in the searobin (*Prionotus carolinus*), a fish with alternate sonic muscle contraction J Exp Biol 2004 207 10 1643 1654 10.1242/jeb.00928 15073197

[B8] Heyd A Pfeiffer W Über die Lauterzeugung der Welse (Siluroidei, Ostariophysi, Teleostei) und ihren Zusammenhang mit der Phylogenese und der Schreckreaktion Rev Suisse Zool 2000 107 165 211

[B9] Ladich F Sound-generating and -detecting motor system in catfish: design of swimbladder muscles in doradids and pimelodids Anat Rec 2001 263 297 306 10.1002/ar.1105 11455539

[B10] Markl H Schallerzeugung bei Piranhas (Serrasalminae, Characidae) J Comp Physiol A 1971 74 1 39 56

[B11] Ladich F Bass AH Sonic motor pathways in piranhas with a reassessment of phylogenetic patterns of sonic mechanisms among teleosts Brain Behav Evol 2005 66 167 176 10.1159/000087157 16088101

[B12] Kastberger G Economy of sound production in piranhas (Serrasalminae, Characidae): I. Functional properties of sonic muscles Zool Jahrb Physiol 1981 85 113 125

[B13] Connaughton MA Fine ML Taylor MH The effect of seasonal hypertrophy and atrophy of fiber morphology, metabolic substrate concetration and sound characteristics of the weakfish sonic muscle J Exp Biol 1997 200 2449 2457 9343856 10.1242/jeb.200.18.2449

[B14] Ono RD Poss SG Structure and innervations of the swimbladder musculature in the weakfish, *Cynoscion regalis* (Teleostei: Sciaenidae) Can J Zool 1982 60 1955 1967 10.1139/z82-253

[B15] Lagardère JP Mariani A Spawning sounds in meagre *Argyrosomus regius* recorded in the Gironde estuary France. J Fish Biol 2006 69 6 1697 1708 10.1111/j.1095-8649.2006.01237.x

[B16] Fine ML Ladich F Kapoor BG, Arratia G, Chardon M, Diogo R Sound production, spine locking and related adaptations Catfishes 2003 Enfield: Science Publishers 248 290

[B17] Parmentier E Diogo R Evolutionary trends of swimbladder sound mechanisms in some teleost fishes Communication in Fishes, Volume 1 2006 Enfield, NH: Science Publishers 45 70

[B18] Skoglund C Functional analysis of swimbladder muscles engaged in sound production of the toadfish J Biophys Biochem Cytology 1961 10 187 200 10.1083/jcb.10.4.187 PMC2225107 19866593

[B19] Crawford JD Huang X Communication signals and sound production mechanisms of mormyrid electric fish J Exp Biol 1999 202 1417 1426 10210682 10.1242/jeb.202.10.1417

[B20] Bass AH McKibben JR Neural mechanisms and behaviors for acoustic communication in teleost fish Prog Neurobiol 2003 69 1 1 26 10.1016/S0301-0082(03)00004-2 12637170

[B21] Amorim MCP Hawkins AD **Ontogeny of acoustic and feeding behaviour in the grey gurnard,** Eutrigla gurnardus Ethology 2005 111 3 255 269 10.1111/j.1439-0310.2004.01061.x

[B22] Sprague MW **The single sonic muscle twitch model for the sound-production mechanism in the weakfish,** Cynoscion regalis J Acoust Soc Am 2000 108 5 2430 2437 10.1121/1.1315296 11108383

[B23] Rauther M Über die Schwimmblase und die zu ihr in Beziehung tretenden somatischen Muskeln bei den Trigliden und anderen Scleroparei Zool Jahrb Anat 1945 69 159 250

[B24] Tavolga WN Mechanism of sound production in the ariid catfish *Galeichthys* and *Bagre* Bull Am Mus Nat Hist 1962 24 1 30

[B25] Lindholm MM Bass AH Early events in myofibrillogenesis and innervation of skeletal sound-generating muscle in a teleost fish J Morphol 1993 216 225 239 10.1002/jmor.1052160209 8515479

[B26] Ladich F Fine ML Localization of swim bladder and pectoral motoneurons involved in sound production in pimelodid catfish Brain Behav Evol 1994 44 86 100 10.1159/000113572 7953611

[B27] Fawcett DW Revel JP The sarcoplasmic reticulum of a fast-acting fish muscle J Biophys Biochem Cytol 1961 10 89 109 10.1083/jcb.10.4.89 13698423 PMC2225096

[B28] Bass AH Marchaterre MA Sound-generating (sonic) motor system in a teleost fish (*Porichthys notatus*): Sexual polymorphisms and general synaptology of sonic motor nucleus J Comp Neurol 1989 286 2 154 169 10.1002/cne.902860203 2794113

[B29] Fine ML Bernard B Harris TM Functional morphology of toadfish sonic muscle fibers: relationship to possible fiber division Can J Zool 1993 71 11 2262 2274 10.1139/z93-318

[B30] Loesser KE Rafi J Fine ML Embryonic, juvenile, and adult development of the toadfish sonic muscle Anat Rec 1997 249 469 477 10.1002/(SICI)1097-0185(199712)249:4<469::AID-AR6>3.0.CO;2-M 9415454

[B31] Schaeffer P Conley K Lindstedt S Structural correlates of speed and endurance in skeletal muscle: the rattlesnake tailshaker muscle J Exp Biol 1996 199 2 351 358 9317944 10.1242/jeb.199.2.351

[B32] Appelt D Shen V Franzini-Armstrong C Quantitation of Ca ATPase, feet and mitochondria in super fast muscle fibers from the toadfish, *Opsanus tau* J Muscle Res Cell Motil 1991 12 543 552 10.1007/BF01738442 1838745

[B33] Ladich F Fine M Ladich F, Collin SP, Moller P, Kapoor BG Sound-generating mechanisms in fishes: a unique diversty in vertebrates Communication in Fishes, Volume 1 2006 Enfield, NH: Science Publishers 3 34

[B34] Rome LC Syme DA Hollingworth S Lindstedt S Maylor SM The whistle and the rattle: the design of sound producing muscles Proc Natl Acad Sci 1996 93 8095 8100 10.1073/pnas.93.15.8095 8755609 PMC38881

[B35] Rome LC Cook C Syme DA Connaughton MA Ashley-Ross M Klimov A Tikkunov B Goldman YE Trading force for speed: why superfast crossbridge kinetics leads to superlow forces Proc Natl Acad Sci 1999 95 5826 5831 10318969 10.1073/pnas.96.10.5826 PMC21945

[B36] Somlyo AV Shurman H Somlyo AP Composition of sarcoplasmic reticulum in situ by electron probe X-ray microanalysis Nature 1977 268 556 558 10.1038/268556a0 887175

[B37] Feher J Waybright T Fine M Comparison of sarcoplasmic reticulum capabilities in toadfish (*Opsanus tau*) sonic muscle and rat fast twitch muscle J Muscle Res Cell Motil 1998 19 6 661 674 10.1023/A:1005333215172 9742450

[B38] Gainer H Kerkut GA Multiple innervation of fish skeletal muscle Experiments in Physiology and Biochemistry, Volume 2 1969 New York, NY: Academic 191 208

[B39] Hirsch JE Bigbee JW Fine ML Continuous adult development of multiple innervation in toadfish sonic muscle J Neurobiol 1998 36 348 356 10.1002/(SICI)1097-4695(19980905)36:3<348::AID-NEU4>3.0.CO;2-W 9733071

[B40] Hamoir G Gerardin-Otthiers N Focant B Protein differentiation of the superfast swimbladder muscle of the toadfish *Opsanus tau* J Mol Biol 1980 143 1 155 160 10.1016/0022-2836(80)90129-1 7441760

[B41] Müller UK van Leeuwen JL Swimming of larval zebrafish: ontogeny of body waves and implications for locomotory development J Exp Biol 2004 207 5 853 868 10.1242/jeb.00821 14747416

[B42] Buss RR Drapeau P Activation of Embryonic Red and White Muscle Fibers During Fictive Swimming in the Developing Zebrafish J Neurophysiol 2002 87 3 1244 1251 11877498 10.1152/jn.00659.2001

[B43] Mauguit Q Olivier D Vandewalle N Vandewalle P Ontogeny of swimming movements in bronze corydoras (*Corydoras aeneus*) Can J Zool 2010 88 4 378 389 10.1139/Z10-012

[B44] Millot S Vandewalle P Parmentier E Sound production in red-bellied piranhas (*Pygocentrus nattereri*, Kner): an acoustical, behavioural and morphofunctional study J Exp Biol 2011 214 21 3613 3618 10.1242/jeb.061218 21993790

[B45] Raamsdonk W Veer L Veeken K Heyting C Pool CW Differentiation of muscle fiber types in the teleost *Brachydanio rerio*, the zebrafish Anat Embryol 1982 164 1 51 62 10.1007/BF00301878 7114488

[B46] Nahirney P Fischman D Wang K Myosin flares and actin leptomeres as myofibril assembly/disassembly intermediates in sonic muscle fibers Cell Tissue Res 2006 324 1 127 138 10.1007/s00441-005-0110-3 16425023

[B47] Eichelberg H Fine structure of the drum Muscles of the Piranha (Serrasalminae, Characidae) Cell Tissue Res 1977 185 547 555 606368 10.1007/BF00220658

[B48] Myrberg AAJ Ha SJ Shamblott MJ The sounds of bicolor damselfish (*Pomacentrus partitus*): predictors of body size and a spectral basis for individual recognition and assessment J Acoust Soc Am 1993 94 3067 3070 10.1121/1.407267

[B49] Lobel PS Mann DA Spawning sounds of the damselfish, *Dascyllus albisella* (Pomacentridae), and relationship to male size Bioacoustics 1995 6 187 198 10.1080/09524622.1995.9753289

[B50] Connaughton M Taylor M Fine M Effects of fish size and temperature on weakfish disturbance calls: implications for the mechanism of sound generation J Exp Biol 2000 203 1503 1512 10751166 10.1242/jeb.203.9.1503

[B51] Colleye O Frederich B Vandewalle P Casadevall M Parmentier E Agonistic sounds in the skunk clownfish *Amphiprion akallopisos*: size-related variation in acoustic features J Fish Biol 2009 75 4 908 916 10.1111/j.1095-8649.2009.02316.x 20738587

[B52] Parmentier E Colleye O Mann D Hearing ability in three clownfish species J Exp Biol 2009 212 2023 2026 10.1242/jeb.030270 19525428

[B53] Lechner W Wysocki L Ladich F Ontogenetic development of auditory sensitivity and sound production in the squeaker catfish Synodontis schoutedeni BMC Biol 2010 8 1 10 10.1186/1741-7007-8-10 20113466 PMC2824629

[B54] Vasconcelos RO Ladich F Development of vocalization, auditory sensitivity and acoustic communication in the Lusitanian toadfish *Halobatrachus didactylus* J Exp Biol 2008 211 502 509 10.1242/jeb.008474 18245626

[B55] Parmentier E Vandewalle P Brie C Dinraths L Lecchini D Comparative study on sound production in different Holocentridae species Front Zool 2011 8 1 12 10.1186/1742-9994-8-12 21609479 PMC3126766

[B56] Wainwright P Richard B Predicting patterns of prey use from morphology of fishes Environ Biol Fishes 1995 44 1–3 97 113

[B57] Van Wassenbergh S Herrel A James RS Aerts P Scaling of contractile properties of catfish feeding muscles J Exp Biol 2007 210 7 1183 1193 10.1242/jeb.000109 17371917

[B58] Bradbury J Vehrencamp S Principles of animal communication 1998 Sunderland, Massachusetts: Sinauer Associates, Inc.

[B59] Hill G Fine M Musick J Ontogeny of the sexually dimorphic sonic muscle in three sciaenid species Copeia 1987 3 708 713

[B60] Onuki A Ohmori Y Somiya H Spinal nerve innervation to the sonic muscle and sonic motor nucleus in red piranha, *Pygocentrus nattereri* (Characiformes, Ostariophysi) Brain Behav Evol 2006 67 2 111 122 10.1159/000089185 16254416

[B61] Veggetti A Mascarello F Scapolo PA Rowlerson A Hyperplastic and hypertrophic growth of lateral muscle in *Dicentrarchus labrax* (L.) Anat Embryol 1990 182 1 1 10 2240590 10.1007/BF00187522

[B62] Rowlerson A Mascarello F Radaelli G Veggetti A **Differentiation and growth of muscle in the fish*****Sparus aurata*****(L): II.** Hyperplastic and hypertrophic growth of lateral muscle from hatching to adult J Muscle Res Cell Motil 1995 16 3 223 236 10.1007/BF00121131 7559995

[B63] Lindstedt SL McGlothlin T Percy E Pifer J Task-specific design of skeletal muscle: balancing muscle structural composition Comp Biochem Physiol Part B Biochem Mol Biol 1998 120 1 35 40 10.1016/S0305-0491(98)00021-2 9787776

[B64] Huriaux F Baras E Vandewalle P Focant B Expression of myofibrillar proteins and parvalbumin isoforms in white muscle of dorada during development J Fish Biol 2003 62 4 774 792 10.1046/j.1095-8649.2003.00064.x

[B65] Focant B Vandewalle P Huriaux F Expression of myofibrillar proteins and parvalbumin isoforms during the development of a flatfish, the common sole *Solea solea*: comparison with the turbot *Scophthalmus maximus* Comp Biochem Physiol Part B Biochem Mol Biol 2003 135 3 493 502 10.1016/S1096-4959(03)00116-7 12831769

[B66] Focant B Huriaux F Vandewalle P Castelli M Goessens G Myosin, parvalbumin and myofibril expression in barbel (*Barbus barbus* L.) lateral white muscle during development Fish Physiol Biochem 1992 10 2 133 143 10.1007/BF00004524 24214210

[B67] Crockford T Johnston IA **Developmental changes in the composition of myofibrillar proteins in the swimming muscles of Atlantic herring,** Clupea harengus Mar Biol 1993 115 1 15 22 10.1007/BF00349381

